# Supplementary material for: Hyperpolarization of Nitrile Compounds Using Signal Amplification by Reversible Exchange
Source: Molecules. 2020 Jul 23;25(15):3347. doi: 10.3390/molecules25153347 (PMC7435364; doi:10.3390/molecules25153347)
Supplement: Supplementary file 1 [file molecules-25-03347-s001.pdf]

Hyperpolarization of Nitrile Compounds Using Signal Amplification by Reversible Exchange

Sarah Kim [1,†], Sein Min [1,†], Heelim Chae [1], Hye Jin Jeong [2], Sung Keon Namgoong [1], Sangwon Oh [3,*] and Keunhong Jeong [2,*]

[1] Department of Chemistry, Seoul Women's University, Seoul 01797, Korea; srh0714@daum.net (S.K.); sein5762@naver.com (S.M.); mek2425@naver.com (H.C.); sknam@swu.ac.kr (S.K.N.)

[2] Department of Chemistry, Korea Military Academy, Seoul 01805, Korea; hyejinj1011@naver.com

[3] Korea Research Institute of Standards and Science, Daejeon 34113, Korea

[*] Correspondence: sangwon.oh@kriss.re.kr (S.O.); doas1mind@kma.ac.kr or doas1mind@berkeley.edu (K.J.); Tel.: +82-2-2197-2823 (K.J.)

[†] These authors contributed equally to this work.

Equation S1. SABRE amplification Calculation

The signal enhancement (ε) were determined by calculating a ratio of the integrals of the reference sample(Ref) and hyperpolarized sample(HP). Here, S_{HP} and S_{Ref} are integral of the signal for the HP and Ref samples, respectively, and C_{HP} and C_{Ref} are the integral of the HP and the Ref solvent peaks of the sample, respectively.

$$\varepsilon = \frac{S_{HP}}{S_{Ref}} \cdot \frac{C_{Ref}}{C_{HP}}$$

Table S1. Concentration (mol%) of Ir-catalyst to nitrile compound for each concentration.

Nitrile Compound Concentration	Acetonitrile	Propionitrile	Butyronitrile	Isobutyronitrile	Valeronitrile
1 μL	14.04	18.35	21.5	22.05	24.69
3 μL	5.16	6.97	8.37	8.49	9.85
5 μL	3.15	4.28	5.2	5.32	6.15
7 μL	2.26	3.11	3.77	3.88	4.47

Table S2. Enhancement of acetonitrile

	1 μL	3 μL	5 μL	7 μL
~0.5 G	24.56776	15.03119	10.22993	10.01882
20 G	52.29815	57.77136	74.93761	107.8079
70 G	57.3087	71.61769	133.5832	115.9412
120 G	41.53924	50.78754	100.6126	103.4024

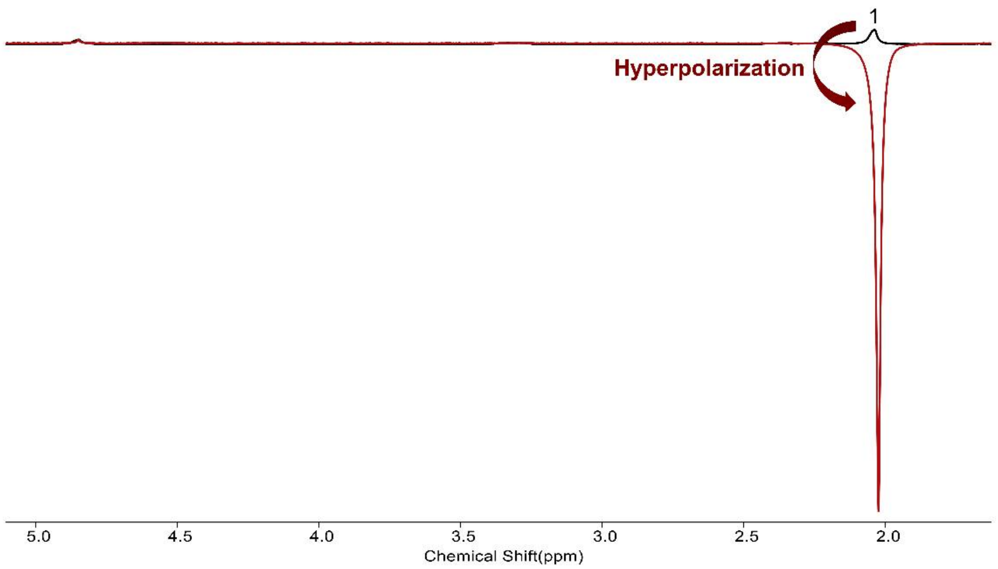

Figure S1. NMR spectrum after SABRE in the 70 G on the 5 μL of acetonitrile. (Ir.cat 3.15 %)

Table S3. Enhancement of propionitrile

	1 μL		3 μL	
	1	2	1	2
~0.5 G	36.71174	27.65089	56.47121	37.16538
20 G	63.73966	5.853063	73.61776	8.218948
70 G	127.39501	23.38615	125.2682	34.37571
120 G	112.63016	34.06832	130.55819	46.09566
	**5 μL**		**7 μL**	
	1	2	1	2
~0.5 G	48.42174	34.04944	45.0767	36.09283
20 G	61.85649	5.300452	72.39131	7.986943
70 G	106.59652	27.88384	97.46813	25.70263
120 G	122.96002	45.00243	107.1576	40.68913

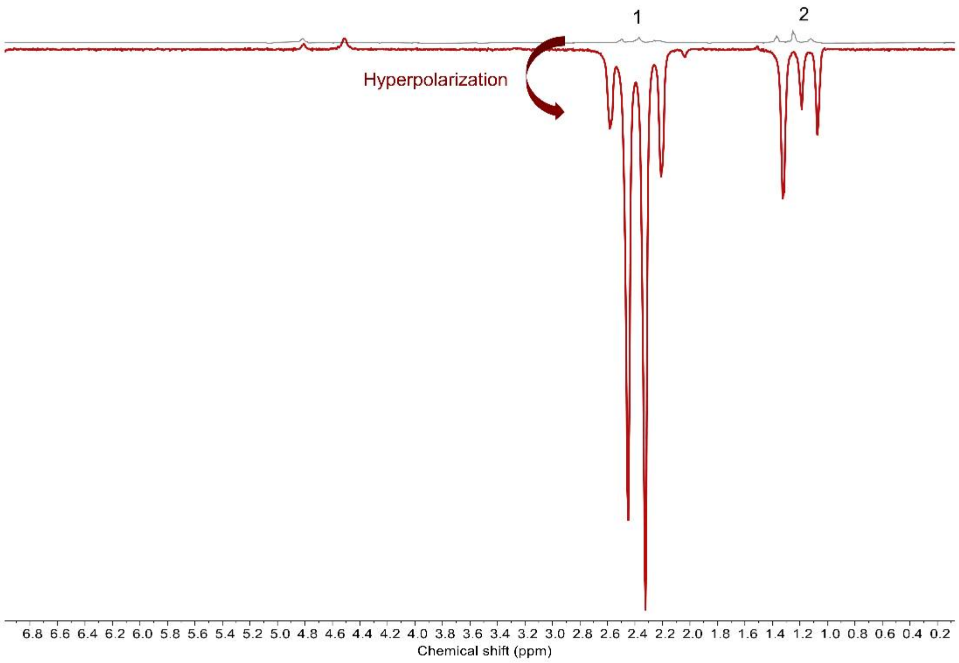

Figure S2. NMR spectrum after SABRE in the 70 G on the 1 μL of propionitrile. (Ir.cat 18.35 mol %)

Table S4. Enhancement of butyronitrile

	1 μL			3 μL		
	1	2	3	1	2	3
~0.5 G	4.32012	8.35794	9.7643	11.82862	6.166202	15.60121
20 G	9.20553	4.39448	3.67458	53.33324	22.86009	16.5569
70 G	7.12852	14.53279	18.0128	47.39608	22.21521	17.84792
120 G	5.70118	16.52928	16.58905	42.32554	23.70027	19.31091
	5 μL			7 μL		
	1	2	3	1	2	3
~0.5 G	35.92597	7.4025	21.77271	21.53454	5.227854	14.91458
20 G	72.53253	31.38602	23.59647	76.61092	28.451	21.0433
70 G	113.5019	50.7516	47.56291	92.8281	36.64095	29.825
120 G	57.22975	32.81679	30.53861	22.08152	16.43635	48.61104

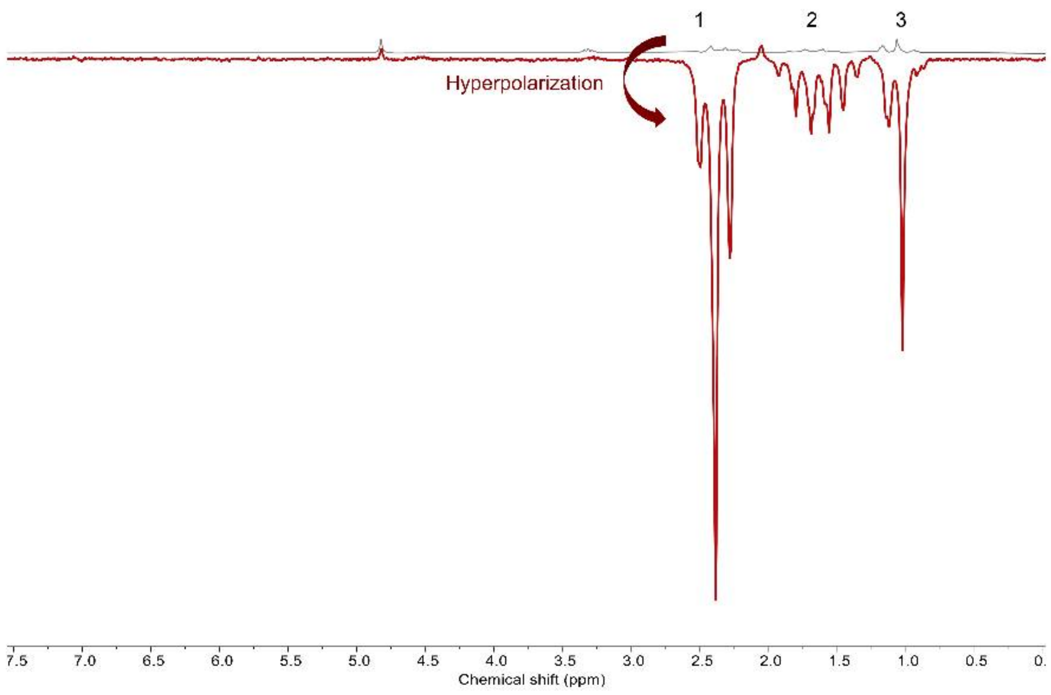

Figure S3. NMR spectrum after SABRE in the 70 G on the 1 μL of butyronitrile. (Ir.cat 21.5 mol %)

Table S5. Enhancement of *iso*butyronitrile

	1 μL		3 μL	
	1	2	1	2
~0.5 G	39.50174	13.25881	46.34633	11.68668
20 G	36.43977	7.458509	55.36512	8.371975
70 G	61.72523	4.002663	41.38677	3.744141
120 G	37.42673	1.630208	34.86906	11.49074
	**5 μL**		**7 μL**	
	1	2	1	2
~0.5 G	65.03643	11.33494	48.99298	11.4661
20 G	103.53445	7.89646	69.51392	2.823158
70 G	90.86874	2.999362	59.53781	0.695038
120 G	80.97548	13.15415	67.49154	14.95109

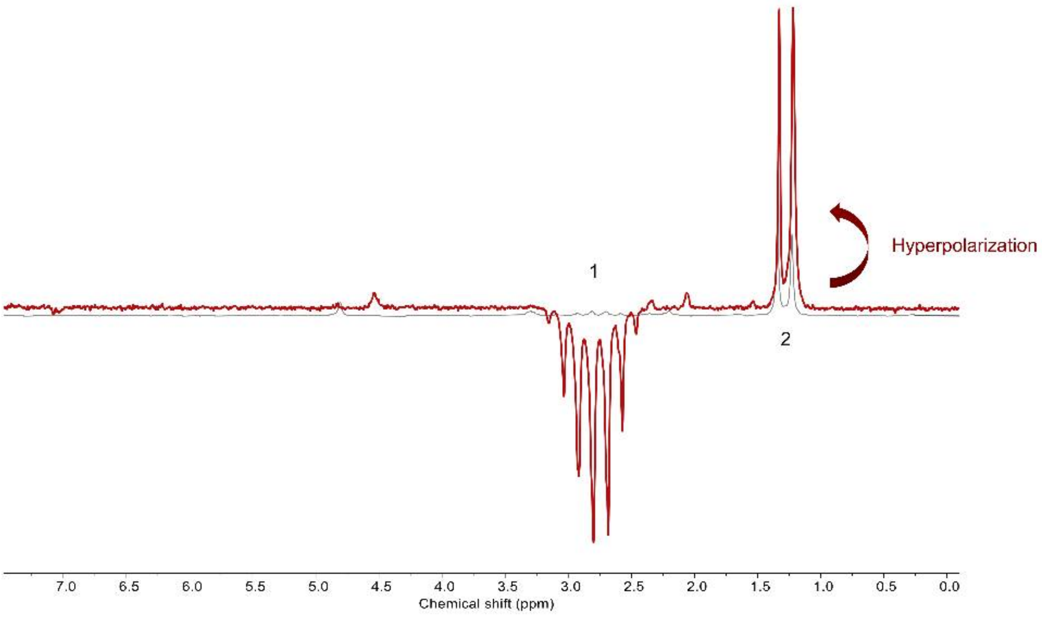

Figure S4. NMR spectrum after SABRE in the 20 G on the 5 μL of *iso*butyronitrile. (Ir.cat 5.32 mol%)

Table S6. Enhancement of valeronitrile

	1 μL			3 μL		
	1	2, 3	4	1	2, 3	4
~0.5 G	2.841543	12.53698	3.058604	51.81755	13.87649	23.82624
20 G	20.8776	21.60702	8.426653	53.3244	33.28789	30.66296
70 G	15.48991	17.73768	13.91461	60.34551	39.41533	39.4968
120 G	60.90311	75.49471	33.42835	45.12613	28.71376	25.19114
	5 μL			7 μL		
	1	2, 3	4	1	2, 3	4
~0.5 G	63.32666	16.93624	27.76323	16.49608	14.13259	19.69747
20 G	103.9058	37.03716	26.26959	53.82877	33.82342	33.43332
70 G	106.3969	57.04372	54.89456	67.89175	42.57545	41.57286
120 G	60.02543	42.33143	41.16961	61.66503	44.78736	43.16849

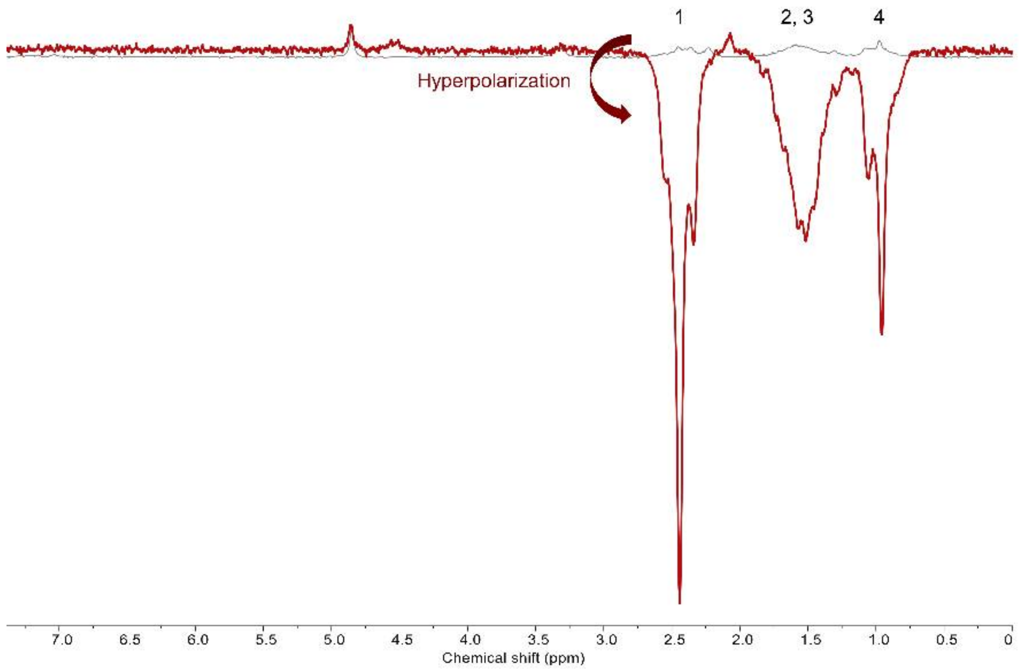

Figure S5. NMR spectrum after SABRE in the 70 G on the 5 μL of valeronitrile. (Ir.cat 6.15 mol%)
